# Adverse Effects of the COVID-19 Pandemic on Movement and Play Behaviours of Children and Youth Living with Disabilities: Findings from the National Physical Activity Measurement (NPAM) Study

**DOI:** 10.3390/ijerph182412950

**Published:** 2021-12-08

**Authors:** Sarah A. Moore, Ritu Sharma, Kathleen A. Martin Ginis, Kelly P. Arbour-Nicitopoulos

**Affiliations:** 1 School of Health and Human Performance, Faculty of Health, Dalhousie University, P.O. Box 15000, Halifax, NS B3H 4R2, Canada; 2 Department of Pediatrics, Faculty of Medicine, Dalhousie University, P.O. Box 15000, Halifax, NS B3H 4R2, Canada; 3 Healthy Populations Institute, Dalhousie University, P.O. Box 15000, Halifax, NS B3H 4R2, Canada; 4 Faculty of Kinesiology and Physical Education, University of Toronto, 55 Harbord St., Toronto, ON M5S 2W6, Canada; ri.sharma@mail.utoronto.ca; 5 School of Health and Exercise Sciences, University of British Columbia, 1147 Research Road, Kelowna, BC V1V 1V7, Canada; kathleen_martin.ginis@ubc.ca; 6 Department of Medicine, Division of Physical Medicine and Rehabilitation, University of British Columbia, 1088 Discovery Avenue, Kelowna, BC V1V 1V7, Canada; 7 International Collaboration on Repair Discoveries (ICORD), University of British Columbia, 818 W 10th Avenue, Vancouver, BC V5Z 1M9, Canada; 8 Centre for Chronic Disease Prevention and Management, University of British Columbia, 1088 Discovery Avenue, Kelowna, BC V1V 1V7, Canada

**Keywords:** children and youth, disability, physical activity, outdoor play, sedentary behaviour, screen time, sleep, parental support, COVID-19, public health restrictions

## Abstract

All children and youth require ample physical activity (PA), low levels of sedentary behaviour (SB), and adequate sleep to stay healthy. Children and youth living with disabilities (CYWD) tend to have fewer opportunities for participation in PA and outdoor play compared with their typically developing peers. In turn, CYWD are typically less active and more sedentary, on average, compared with their peers. The COVID-19 pandemic reduced opportunities for many children and youth to participate in PA and outdoor play. The purpose of this study was to assess parent-perceived changes in PA (including outdoor play), SB (including screen time), and sleep quality and quantity, due to COVID-19 and related restrictions, in a national cohort of Canadian CYWD. We recruited parents of school-aged CYWD (*N* = 151) in May 2020. In an online survey, parents reported their child’s previous 7-day PA, SB, and sleep, as well as perceived changes in their child’s movement and play behaviours due to COVID-19 and related restrictions. Parent-perceived parental support for their child’s movement and play behaviours during the pandemic was also assessed. We used descriptive statistics to describe the child’s movement behaviours and assessed the association between movement behaviours and parental factors using Pearson and point-biserial correlations. Few (5.3%) CYWD met PA recommendations and 13.2% met screen time recommendations during the acute period of the COVID-19 pandemic. More CYWD (66.2%) were meeting sleep recommendations. Overall, only 1.3% of CYWD were meeting the combined movement guidelines. Parent encouragement was positively associated with the child’s outdoor PA (0.23), wheeling, walking, and biking (0.19), indoor PA (0.16), and family-based PA (0.26). Parental co-play was similarly positively associated with the child’s outdoor PA (0.26), wheeling, walking, and biking (0.39), indoor PA (0.16), and family-based PA (0.26). Parents perceived their CYWD to be less active and more sedentary as a result of COVID-19 and the related restrictions. Parents of CYWD have an important role in encouraging healthy movement behaviours. Return to movement and play post-COVID guidelines should include tailored strategies for CYWD and their families to mitigate the negative impacts of the pandemic.

## 1. Introduction

The benefits of physical activity (PA) are universal for all children and youth, including children and youth living with disabilities (CYWD). Children and youth who engage in regular daily PA tend to have better physical [1] and mental [2] health compared with their less-active peers. Further, children and youth who meet daily PA recommendations typically engage in less sedentary behaviour (SB) and screen time [3] and sleep better [4] compared with children and youth who do not meet the recommendations. Combined, sufficient PA, reduced SB, and adequate sleep (i.e., the 24-hour movement behaviours [5,6]) are important health behaviours for all children and youth, and have been recognized by health-promoting organizations as necessary contributors to healthy childhood growth and development. However, despite the known benefits, the majority of Canadian children and youth do not accumulate enough daily PA and sleep, nor limit their SB enough to meet the guidelines. For example, prior to the COVID-19 pandemic, only 12.7% and 17.1% of Canadian children and youth were meeting the PA recommendations using parent-reported and device-based measures, respectively [1,7].

In Canada, 5% of children under 15 years and 13% of youth aged 15 to 24 years live with a disability [8,9]. CYWD tend to participate less in PA and sport [10,11,12], engage in more SB including excess screen time [13,14,15], and have more disrupted sleep [16,17,18] compared with their typically developing peers. There is a lack of reliable evidence on the movement behaviours of CYWD, and there are no population level data on movement behaviours of Canadian CYWD [19,20]. The National Physical Activity Measurement (NPAM) study [21] launched in 2018 with the aim of assessing PA, SB, and sleep in Canadian CYWD. This ongoing study aims to produce the first Canadian population-level data for school-aged children and youth with all types of disabilities on rates of PA, SB, and sleep, establish demographic and health profiles for those CYWD who meet and do not meet the 24-h movement guidelines, and describe different types and durations of activities being undertaken by CYWD. In March 2020, NPAM data collection was disrupted when the World Health Organization (WHO) declared COVID-19 a global pandemic [22].

To curb the transmission of the first wave of COVID-19, several public health restrictions were imposed, such as school closures and childcare closures, cessation of organized physical activities and sports, and limited access to outdoor play spaces, such as playgrounds and parks, as well as new policies such as masking and physical distancing [23]. The restrictions had unprecedented impacts on the lives of children and youth [24] and limited how children and youth engaged in healthy movement and outdoor play. Studies assessing the changes in movement behaviours of children and youth during COVID-19 have consistently reported declines in PA, increases in SB, and changes in sleep [25]. In a national study of typically developing Canadian children and youth, only 2.6% were meeting the 24-h movement guidelines during the first wave (April 2020) of the pandemic [26]. This study was the first to note the potential collateral consequences of the COVID-19 pandemic on child health and health-related behaviours.

While there is growing evidence on the impact of the pandemic on children’s and youth’s movement behaviours, very few studies have included or focused specifically on CYWD. In a recent scoping review that identified 150 studies on childhood movement behaviours during the first year of the COVID-19 pandemic [25], only 4 empirical studies [27,28,29,30] and 2 commentaries [31,32] focused on movement or play behaviours of CYWD. The ECHO French survey assessed emerging health challenges of children and youth living with physical disabilities (e.g., cerebral palsy, neuromuscular diseases) and found that 44% of CYWD stopped participating in physical activities due to the pandemic and public health restrictions [27]. Esentürk [28] interviewed 10 parents of children living with autism spectrum disorder (ASD). Parents felt that time spent in PA enhanced their child’s psychosocial health during the pandemic. Garcia et al. [29] also sampled a cohort of parents of children with ASD. The survey showed that the number of days children were meeting PA recommendations declined by half and screen time increased by one-third. Masi et al. [30] found more extensive declines in PA amongst a cohort of children living with neurodevelopmental/developmental disabilities, where 68% of CYWD (specifically children living with neurodevelopmental disabilities) were exercising less. Further, 81.6% of parents of CYWD reported that their child had increased their screen time (e.g., television and digital media) and reduced their sleep quality (43.6%) [30]. Although there is emerging research on the pandemic’s effect on the movement and play behaviours of typically developing children and youth, there is a need for more evidence on how the pandemic and related restrictions have impacted CYWD. The COVID-19 pandemic has isolated children and youth [33], reduced their engagement in healthy behaviours [26,34], and has led to childhood traumatic stress and mental health issues [35,36]. COVID-19 threatens to exacerbate health disparities among persons with disabilities, including healthy movement and play behaviours in CYWD [37]. Canadian parents of CYWD are worried about their child’s physical and mental health as a result of the pandemic, concerned about their child being socially isolated, and concerned that their child would not participate in adequate amounts of PA to support their health during the pandemic [38]. Engaging in play and PA may help to mitigate the negative effects of the COVID-19 pandemic [39,40]. This is also true for CYWD [41]. Thus, as we recover from the COVID-19 pandemic, there is a need to better understand the factors that may support or limit participation in healthy movement and play behaviours during the pandemic.

Parental support has been shown to be a key determinant of healthy movement and play behaviours in CYWD [42,43,44]. Parental support may include tangible support (e.g., co-participation in healthy behaviours, providing transportation to PA programs or services) or intangible support (e.g., encouraging healthy behaviours or discouraging unhealthy behaviours) [45]. Healthy childhood behaviours can be facilitated by parents who feel capable and have the opportunity to support their child’s healthy behaviours [46]. These factors are even more critical for CYWD, who may require additional supports to adequately participate in healthy movement and play behaviours [47,48]. Previous studies that assessed the relationship between movement and play behaviours and parental factors in Canadian children and youth showed that parental support and encouragement (e.g., co-participation) were associated with healthy childhood behaviours [26,34]; however, these relationships have not yet been explored in CYWD during the COVID-19 pandemic.

The purpose of this study was to describe the movement and play behaviours of a national sample of Canadian CYWD during the first wave of the COVID-19 pandemic (May 2020). We aimed to (a) describe the parent-perceived changes in their child’s movement and play behaviours attributed to the COVID-19 pandemic, and (b) examine parental support (e.g., parental support and encouragement, parental-perceived capability, and the opportunity to support healthy behaviours) correlates of movement and play behaviours during the COVID-19 pandemic. We hypothesized reduced PA, increased SB, and increased sleep in CYWD during the COVID-19 pandemic. We also hypothesized that parental support behaviours would be correlated with their child’s healthy movement and play behaviours. As one of the few studies to examine movement and play behaviours in CYWD during the COVID-19 pandemic, we expect these study findings will describe the deleterious effects of COVID-19 on movement behaviours of CYWD and their families during the pandemic. We also anticipate our results will inform strategies to support the health of CYWD during the recovery from this global health crisis.

## 2. Materials and Methods

### 2.1. Study Design and Sample

Study participants were recruited from the National Physical Activity Measurement (NPAM) study cohort. In April and May 2020, approximately one month after the WHO’s declaration of COVID-19 as a global pandemic, we recruited parents of school-aged CWYD (*N* = 151) who had previously participated in the NPAM study. Parents were eligible to participate in the study if they participated in the NPAM study, over the age of 18 years, had a CYWD, and were English speaking. Parents provided informed consent at the beginning of the online survey after reading a letter of information and consent form. The study followed the principles of the World Medical Declaration of Helsinki and was approved by the Research Ethics Board of the University of Toronto (#31862).

### 2.2. Survey Development

This study used an amended version of the 2020 COVID-19 and Childhood Movement Behaviours Survey (Appendix A; Table A1), developed by Moore et al. [26]. This survey was previously used to assess the movement and play behaviours of Canadian children and youth during the COVID-19 pandemic. The survey was developed by applying a social ecological framework to consider variables at the child, family, and community levels [49]. Child and parent demographic variables included in the survey were child age, gender, disability, and health status; as well as parental age, gender, ethnicity, education, marital status, current level of parental distress, income (household), and dwelling type. Child movement behaviour variables included current levels of PA, SB, and sleep, adapted from the Canadian Health Measures Survey [50], and parent-perceived changes in their child’s movement and play behaviours as a result of COVID-19 and related restrictions. Parental support survey variables included parent-perceived changes in encouragement and support of PA, outdoor play, and sleep, and discouragement of SB resulting from the COVID-19 pandemic and related restrictions. Parents were also asked about their capability and opportunity to support PA, outdoor play, and sleep and to discourage SB during COVID-19. Parent support items used a 5-point Likert-type scale, ranging from ‘a lot less’ (score = 1) to ‘a lot more’ (score = 5). If parents perceived their child to be doing the same amount of the behaviour as before the COVID-19 pandemic (i.e., that their child’s behaviour had not changed as a result of the pandemic) the parent selected a score of 3 (‘no change’). Previous analysis demonstrated that the survey showed strong test-retest reliability [26].

### 2.3. Survey Distribution and Data Collection

Parents who had previously participated in the NPAM study (*N* = 495) were sent an email describing the sub-study and invitation to participate in April and May 2020. Interested parents were directed to a secure, data-encrypted website to complete the study using REDCap^®^ (response rate = 31%). Parents were asked to complete the survey within three weeks of receiving the invitation to participate. After reviewing the information letter and providing informed consent, parents completed the online survey (which took approximately 20 min to complete). If a parent had more than one CYWD, the parent could choose to complete the survey more than once (i.e., one survey per child). After data collection was complete, the data were cleaned and prepared for analysis.

### 2.4. Statistical Analysis

Data were analyzed in SPSS 23 (SPSS Inc, Chicago, IL, USA). Descriptive statistics (means, standard deviations) were calculated for the total sample, and by age group and gender. Factorial analyses of variance (by age group and gender) were used to test for differences between continuous variables, and Chi-square tests to test for differences between categorical variables. Statistical significance was set at *p* < 0.01. The proportions of CYWD meeting the Canadian 24-h movement guidelines were determined [5]. To create a visual representation of the changes in movement and play behaviours as a result of COVID-19 and related restrictions, means and standard errors were plotted for selected variables (changes in PA, SB, sleep, and overall healthy behaviours). Pearson and point-biserial correlations were used to determine associations between the movement and play behaviours of CYWD and parental demographic factors and parental support variables.

## 3. Results

### 3.1. Participants

A total of 151 NPAM parents of CYWD (ages 4 to 17 years) completed the survey. Table 1 includes descriptive statistics for parent and child characteristics. Respondents were primarily women (92.1%), married or cohabiting (84.1%), college or university graduates (78.1%), and worked outside of the home in a full- or part-time capacity (78.1%). Children and youth (mean age = 10.77 years) were living with either a developmental (e.g., ASD, 37.1%), physical (e.g., spinal muscular atrophy, 13.9%), or sensory disability (e.g., visual impairment, 4.0%), or more than one disability (45.0%). Most children and youth were boys (74.8%). The type of home was predominantly a detached or semi-detached home (82.1%), and the average number of adults and children in the household were 2.05 and 2.19, respectively. When parents were asked about how their child’s health changed during the COVID-19 pandemic, 17% of parents reported a decline in their child’s health status.

### 3.2. Movement and Play Behaviours in Children and Youth Living with Disabilities

A summary of the movement behaviours of CYWD is presented in Table 2. While the majority of CYWD met sleep recommendations for their age (66.2%), most did not meet the MVPA or SB (screen time) guidelines (5.3% and 11.3%, respectively). Overall, only 1.3% of CYWD met the combined 24-h movement guidelines (MVPA, SB (screen time), and sleep). No youth (14–17 years of age) met the individual MVPA or SB (screen time) guidelines or the combined 24-h movement guidelines.

**Figure 1:**
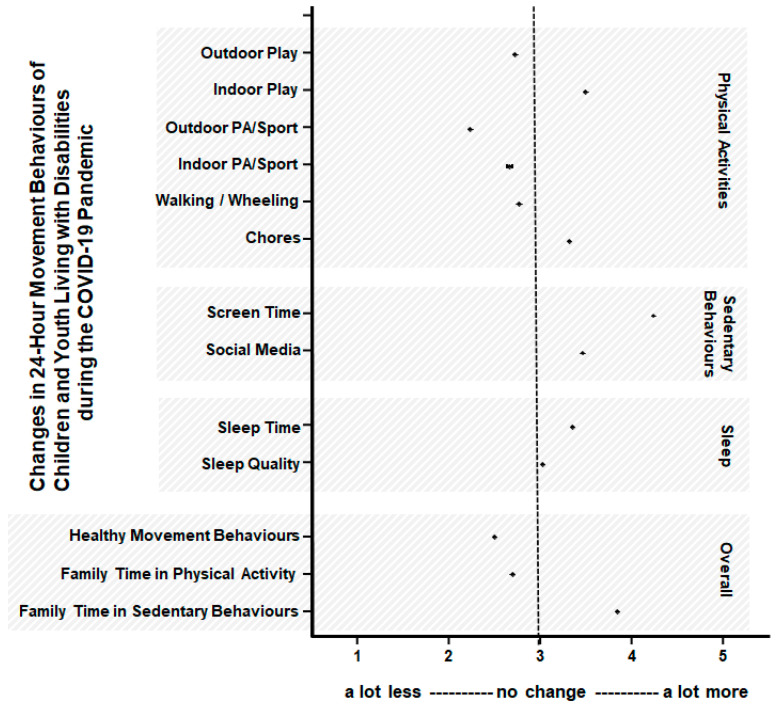
Parent-reported changes in 24-h movement behaviours in Canadian children and youth (5 to 17 years) living with disabilities (CYWD) during the COVID-19 pandemic (May 2020). Scores are based on a 5-point scale range from ‘a lot less’ (score 1) to ‘about the same’ (score 3) to a lot more (score 5). See Table 2 and Table 3 for more details. Data points are means with standard error bars (too small to be seen).

Parent-perceived changes in their child’s movement and play behaviours are presented in Table 3. Overall, parents reported a decline (i.e., score < 3) in their child’s outdoor and indoor physical activities (except for an increase in household chores), increase (i.e., scores >3) in their child’s sedentary behaviours (e.g., screen time, social media), and slight increase (i.e., score >3) in sleep quantity. Most results did not differ significantly by age or gender, except parents of youth reported more of an increase in household chores and sleep quantity compared with parents of children (*p* < 0.05). Figure 1 presents an illustration of the overall parent-perceived changes in their child’s PA, SB, and sleep during the COVID-19 pandemic (May 2020).

### 3.3. Asscoations between Parental Demographics and Child Movement and Play Behaviours

A summary of the associations between parental demographic factors and their child’s movement and play behaviours is presented in Table 4. Briefly, related to parental demographic factors, for those associations where r > 0.1 and *p* < 0.01, we found that living in a two-parent household (i.e., married or cohabiting parents) was associated with higher levels of child and youth outdoor PA (r = 0.27), outdoor play (r = 0.26), and family-based PA (r = 0.26). Living in a detached home was also associated with more child time spent in outdoor play (r = 0.28).

### 3.4. Changes in Parental Support Variables during COVID-19

Parent-perceived changes in their support behaviours of their child’s movement and play behaviours as a result of the COVID-19 pandemic are reported in Table 5. Overall, parents reported an *increase* in how much support and encouragement (i.e., intangible supports) they were providing their child (i.e., score > 3) to participate in PA, reduce SB and screen time, and getting adequate sleep. However, parents reported a *decrease* (i.e., score <3) in the amount of time spent driving their child to PA and sport (i.e., tangible supports). Parents responded that their capability and opportunity to support PA and sleep *increased* (i.e., score > 3); however, their capability and opportunity to support healthy amounts of SB (namely screen time) *decreased* (i.e., score < 3) as a result of the COVID-19 pandemic.

### 3.5. Associations between Parental Support and Child Movement and Play Behaviours

A summary of the associations between parental support and child movement and play behaviours is presented in Table 6. Briefly, related to parental support, for those associations where r > 0.1 and *p* < 0.01, we found that parent encouragement of child and youth’s PA and parent co-participation in PA was associated with more child time spent outdoors in PA (r = 0.24 and r = 0.26, respectively) and outdoor play (r = 0.26 and r = 0.34, respectively). Parent encouragement of child and youth’s PA and parent co-participation in PA was associated with increased child time spent in family-based PA (r = 0.26 and r = 0.41, respectively). Parent encouragement of child and youth’s household chores was associated with more household chores (r = 0.21); however, parents driving their child to PA was associated with fewer chores (r = –0.25). The strengths of these associations were small to moderate [51].

Parent capacity and opportunity to support PA was associated with more child outdoor PA (r = 0.29 and r = 0.29, respectively), play (r = 0.23 and r = 0.31, respectively), walking, wheeling, and cycling (r = 0.23 and r = 0.18, respectively), family-based PA (r = 0.39 and r = 0.35, respectively), and overall healthier movement behaviours (r = 0.23 and r = 0.37, respectively). Parent capacity and opportunity to reduce their child’s SB (i.e., screen time) was associated with more child outdoor PA (r = 0.42 and r = 0.35, respectively), play (r = 0.33 and r = 0.29, respectively), walking, wheeling, and cycling (r = 0.28 and r = 0.22, respectively), family-based PA (r = 0.22 and r = 0.18, respectively), and reduced screen time (r= −0.30 and r= −0.27, respectively). Parent capacity and opportunity to support their child’s sleep was associated with better sleep quality (r = 0.39 and r = 0.20, respectively) and family-based PA (r = 0.41 and r = 0.21, respectively). The strengths of these associations were small to moderate [51].

## 4. Discussion

### 4.1. Summary of Findings

This study highlights the detrimental effects of the COVID-19 pandemic and related public health restrictions on the movement and play behaviours of a national sample of Canadian CYWD. Only 1.3% of CYWD met the Canadian 24-h Movement Guidelines for Children and Youth [5] during the acute initial phase (May 2020) of the COVID-19 pandemic. Overall, CYWD were less active and on their screens more during the pandemic. Parents reported declines in their child’s health. Parent-perceived capability and opportunity to support their child’s healthy movement and play behaviours were positively and moderately associated with increased movement and play behaviours. For example, parent capability and opportunity to support their child’s PA was associated with increased outdoor PA and family-based PA. Parent capability and opportunity to support their child’s reduced SB was associated with reduced screen time, more outdoor PA, and more family-based PA. Parent capability and opportunity to support healthy amounts of sleep was associated with their child sleeping longer and increased sleep quality. This study adds to the limited evidence of the pandemic’s consequences on the health behaviours of CYWD. It showcases the role of parents in supporting the movement and play behaviours of the CYWD to mitigate the collateral consequences of the COVID-19 pandemic.

### 4.2. COVID-19 and Related Restrictions Exacerbated Barriers to Movement and Play Declined

The COVID-19 pandemic brought major upheavals in the lives of CYWD. The findings from this study were consistent with other studies that demonstrated a reduction in PA and increase in SB during the pandemic in CYWD [27,28,29,30]. Traditional barriers to healthy movement and play that CYWD experience may be categorized into individual factors (e.g., fear, pain), sociocultural factors (e.g., family and friend support), environmental factors (e.g., built environment, weather) and/or systemic factors (e.g., inaccessibility facilities) [42,44]. During the COVID-19 pandemic, participation in PA and play has been further challenged and barriers have worsened. For example, CYWD and their families may be experiencing increased fear of participation given the risk of COVID-19 infection. In our study, using the distress thermometer [52,53], parents reported moderate to high levels of distress. Parental distress was associated with reduced outdoor physical activities, reduced outdoor play, and reduced family-based PA in CYWD. In a study of mothers of CYWD, mothers reported that the pandemic heightened their fears, especially that they were fearful of their child becoming ill and that their medical complexities could be made worse if infected with COVID-19. As a result, these parents felt that ordinary daily activities (like PA and play) were now considered hazardous [54]. It is possible that the parents in our study had similar concerns over their child’s health, promoting further precautions and limiting their engagement in PA and play.

Parents of CYWD may have had limited supports during the pandemic. For example, the pandemic may have limited CYWD’s supports from schools, friends, and programs. The restrictions at the time of this survey were extensive in many places in Canada. Most children and youth were not attending in-person classes and recreational facilities were closed and sports programs were ceased [55]. Parents of CYWD could be left feeling as though they do not have the adequate resources to support their child’s PA and play pursuits. In our study, parents reported that their child’s most common playmate before the pandemic was their friends, and during the pandemic was their siblings or a parent. During the pandemic, the play spaces that CYWD were able to access changed; no longer were play spaces schools and gym facilities, but instead inside the home and potentially outdoors. CYWD previously identified the importance of gym settings, during and after-school programs, and outdoor play spaces in supporting their healthy movement behaviours [56]. As such, these closures had implications for participation in PA and play for children and youth, which were undoubtably exacerbated for CYWD and their families. While there are advances in the accessibility of outdoor spaces for people living with disabilities in Canada (e.g., [57]), many outdoor spaces are still not adequately inclusive. Moreover, equipment to access the outdoor for CYWD is expensive, and availability is low given the demand due to the pandemic, including rental programs where turnover is slower and there are increased sanitization protocols. Further, rehabilitation services, such as physical, occupational, and recreation therapy, are considered vehicles to promote healthy movement and play in CYWD, but during the pandemic, many rehabilitation services for people with disabilities were discontinued, shifting to virtual or telerehabilitation platforms [58]. Overall, parents of CYWD had substantially fewer options for support during the pandemic.

### 4.3. Parent Support Contributes to Healthy Movement Behaviours

The sudden and unexpected changes caused by the COVID-19 pandemic and public health restrictions created new difficulties for CYWD and their families. Caring for a child living with a disability was *already* associated with increased parental stress and some parents with CYWD previously reported increased depressive symptoms and anxiety compared with parents of typically developing children [59]. However, and importantly, families of CYWD are also known for their resilience [60], particularly when parents report high self-efficacy [61]. Our study assessed the changes in parental support variables (encouragement, support, capability, opportunity) during the COVID-19 pandemic and their associations with healthy movement behaviours. Parents reported that they encouraged and supported their child’s movement behaviours more during the pandemic. This may be explained by the transition for these children and youth from school to home. Parents and siblings became the CYWD’s primary playmates. We found associations between parent-perceived capability and opportunity to support their child’s movement and play behaviours and increased movement and play behaviours. Bassett-Gunter et al. [46] conducted focus groups with parents of CYWD and found that parent self-efficacy and self-regulatory strategies were important in supporting their child’s healthy movement. Perhaps considering strategies to promote parental capability and opportunity would enhance overall family movement behaviours.

Promoting healthy movement happens in various settings (e.g., at home, at school, within the community). However, during the pandemic lockdown, children and youth spent significantly more time in their homes with their families. Evidence of parental and family influence of child PA, SB, and sleep were so compelling that last year a consensus statement was released on the role of the family in movement behaviours of children and youth aged 0–17 years [62]. The statement describes the contributions of modelling [63] and co-participation [64] in encouraging children to be active and play. Aligning with this evidence, we found a positive association between parental co-participation and many movement and play behaviours. Consequent to the pandemic, these family support behaviours are conceivably even more important during lockdown and as we begin to re-engage in movement post COVID-19. Several studies have, however, shown that parents of CYWD often experience their own barriers to being active (e.g., [65,66,67,68]). Future studies may wish to assess the relationship between child and parent movement and play behaviours and consider interventions to support families moving and playing together, particularly in the context of COVID-19.

### 4.4. Returning to Healthy Movement and Play

In our study, parents reported that their child’s physical and mental health declined as a result of the pandemic and related public health restrictions. This is alarming as CYWD are already at risk for secondary physical (e.g., cardiovascular disease, obesity) and mental (e.g., anxiety, loneliness) health conditions [69,70,71]. The COVID-19 pandemic may heighten these risks for CYWD. Attempts to return to PA and sport have varied during subsequent waves of the pandemic. Enhancing PA and play for CYWD should be considered a priority to support physical and mental health as we recover from the COVID-19 pandemic. Enhancing opportunities for PA and play as we recover from the pandemic may also mitigate some of the negative health consequences CYWD have experienced. PA and play support physical and mental health in all children and may support coping with difficulties associated with the pandemic and enhance resilience [39,41].

Increasing PA and reducing SB in CYWD is complex, particularly when there are public health restrictions due to a pandemic. There are limited recommendations related to return to movement and play that include CYWD. Uniquely, the Ottawa Return to Play Roadmap [72] includes resources and documents to help support the safe return to play for people living with disabilities. Some resources include sanitization protocols for adaptive equipment and including support staff when the number of attendees at programs are limited. More resources are needed to support CYWD and to advocate for the needs of these families. The impact of reduced PA, increased SB, and disrupted sleep on health for CYWD will depend on the duration of this pandemic and its public health restrictions and the inclusiveness of return to PA and play initiatives.

### 4.5. Strengths, Limitations, and Future Directions

This study contributes to the limited but growing literature on how the COVID-19 pandemic has changed the daily lives of CYWD and their families. This study recruited participants from the NPAM study, and as such included a national sample of parents of CYWD from across Canada. The cohort represented a diverse group of Canadian children and youth living with physical, sensory, and neurodevelopmental disabilities. Although the survey was previously deemed reliable [26], it was adapted for use in our population, and may also be subject to social desirability or recall biases. Our study was cross-sectional and would be enhanced by following up with this cohort again through the NPAM study. This approach would allow for better understanding of the long-term consequences of the pandemic and public health restrictions on the movement and play behaviours of CWYD and their families.

## 5. Conclusions

This study provides evidence of the collateral consequences of the COVID-19 virus outbreak and related public health restrictions on CYWD and their families. The results demonstrate that most CYWD were not engaging in sufficient healthy movement behaviours during the pandemic. Given the importance of engaging in movement behaviours for CYWD, it is critical that return to PA plans are inclusive and accessible for all children and youth. Our study adds additional evidence demonstrating that CYWD are experiencing declines in health as a result of the pandemic and related restrictions and highlights the important role that parents play in supporting their child’s healthy movement and play. Our study indicates that it is important that parents of CYWD feel they have the capability and opportunity to support their child’s healthy movement and play. We anticipate our study’s findings can support return to movement and play recommendations and guide efforts to mitigate potential health risks to CYWD and their families during future pandemics.

## Figures and Tables

**Table 1:** Parent, child, and youth characteristics (*N* = 151).

**Parent Demographic Profile**	
Age (years), *M* (SD)	42.42 (5.74)
Gender, woman, *n* (%)	139 (92.1)
Ethnicity, *n* (%)	
European	114 (75.5)
Asian	24 (15.9)
Indigenous	3 (2.0)
Other	10 (6.6)
Marital status, *n* (%)	
Married or common-law	127 (84.1)
Divorced or separated	9 (6.0)
Single	13 (8.6)
Undisclosed	2 (1.3)
Education, *n* (%)	
High school or less	8 (5.3)
College or technical school	58 (38.4)
University	60 (39.7)
Advanced degree	23 (15.3)
Undisclosed	2 (1.3)
Annual household income, *n* (%)	
<$50,000CDN	35 (23.2)
$50,000 to 99,999CDN	41 (27.1)
>$100,000CDN	60 (39.7)
Undisclosed	15 (10.0)
Employment status, *n* (%)	
Full-time	69 (45.7)
Part-time	29 (19.2)
Self-employed	20 (13.2)
Unemployed	28 (18.6)
Other (e.g., student, retired)	5 (3.3)
**Child Demographic Profile**	
Age (years), *M* (SD)	10.77 (3.22)
Ages 4 to 13 years (years), *M* (SD) / *n* (%)	9.62 (2.45)/121 (80.13)
Ages 14 to 17 years (years), *M* (SD) / *n* (%)	15.40 (0.90)/30 (19.87)
Gender, girl, *n* (%)	38 (25.2)
Disability type, *n* (%)	
Developmental	56 (37.1)
Physical	21 (13.9)
Sensory	6 (4.0)
Combination/Other	68 (45.0)
Child’s residence type, *n* (%)	
House (detached or semi-detached)	124 (82.1)
Apartment or townhome	27 (17.9)
Household makeup, *M* (SD)	
Adults	2.05 (0.64)
Children and youth	2.19 (0.96)

**Table 2:** Parent-reported movement behaviours of children (5–13 years) and youth (14–17 years) and proportion meeting the Canadian 24-h Movement Guidelines for Children and Youth during the acute phase (May 2020) of the COVID-19 pandemic (*N* = 151).

	Total	Children	Youth	Boys	Girls
	*N* = 151	*N* = 121	*N* = 30	*N* = 113	*N* = 38
**Current Movement Behaviours of Children and Youth, *M* (SD)**					
MVPA **^1^** ≥ 60 min/day (days/week)	1.60 (2.05)	1.82 (2.16)	0.73 (1.20)	1.56 (2.07)	1.74 (2.01)
Screen time (hours/day)	5.36 (3.32)	5.08 (3.22)	6.49 (3.50)	5.72 (3.31)	4.29 (3.12)
Sleep (hours/day)	9.33 (1.53)	9.39 (1.52)	9.13 (1.59)	9.26 (1.50)	9.58 (1.64)
**Proportion of Children and Youth Meeting Movement Guideline, %**					
MVPA	5.3	6.6	0.0	5.3	5.3
Screen time	11.3	14.0 ^a^	0.0	7.1	23.7 ^g^
Sleep	66.2	66.9	63.3	69.0	57.9
24-h movement behaviours **^2^**	1.3	1.7	0.0	1.0	2.6

^1^ MVPA = moderate-to-vigorous physical activity; ^2^ Includes physical activity, sleep, and screen time; a = significant age difference, g = significant gender difference (*p* < 0.05).

**Table 3:** Parent-reported changes in movement and play behaviours of children (5–13 years) and youth (14–17 years) during the acute phase (May 2020) of the COVID-19 pandemic (*N* = 151).

	Total	Children	Youth	Boys	Girls
	*N* = 151	*N* = 121	*N* = 30	*N* = 113	*N* = 38
**Change in Child Movement and Play Behaviours During COVID-19 Outbreak, *M* (SD) ^1^**					
**Outdoor Physical Activity**					
Outdoor physical activity or sport	2.24 (1.39)	2.24 (1.41)	2.23 (1.33)	2.18 (1.35)	2.42 (1.54)
Outdoor play	2.70 (1.34)	2.68 (1.38)	2.80 (1.16)	2.68 (1.32)	2.76 (1.38)
Walk, wheel, or bike in the neighbourhood	2.74 (1.49)	2.74 (1.50)	2.73 (1.46)	2.68 (1.47)	2.92 (1.53)
**Indoor Physical Activity**					
Indoor physical activity or sport	2.45 (1.23)	2.45 (1.22)	2.45 (1.30)	2.38 (1.19)	2.63 (1.36)
Indoor play	3.51 (1.13)	3.56 (1.10)	3.28 (1.22)	3.48 (1.13)	3.59 (1.12)
Household chores	3.40 (0.90)	3.32 (0.93)	3.70 ^a^ (0.70)	3.43 (0.91)	3.29 (0.87)
**Sedentary Behaviours**					
Television (TV) or other screens	4.30 (0.98)	4.28 (0.99)	4.37 (0.96)	4.36 (0.97)	4.11 (0.98)
Social media	3.25 (1.06)	3.22 (1.09)	3.40 (0.93)	3.24 (1.07)	3.29 (1.04)
Non-screen-based leisure activities	3.60 (0.99)	3.60 (1.01)	3.63 (0.89)	3.57 (0.99)	3.74 (0.98)
**Sleep**					
Sleep quantity	3.20 (0.87)	3.09 (0.86)	3.60 ^a^ (0.81)	3.12 (0.77)	3.43 (1.09)
Sleep quality	3.02 (0.90)	3.00 (0.90)	3.10 (0.92)	3.02 (0.86)	3.03 (1.03)
**Family-Based Activity**					
Physical activity	2.87 (1.24)	2.86 (1.25)	2.90 (1.24)	2.79 (1.27)	3.08 (1.12)
Sedentary behaviours	3.83 (0.98)	3.81 (1.01)	3.90 (0.88)	3.85 (0.97)	3.76 (1.05)
**Overall Healthy Movement Behaviours**	2.49 (1.03)	2.47 (1.02)	2.57 (1.10)	2.45 (1.04)	2.61 (1.03)

^1^ Ranges from 1 to 5, where 1 = a lot less, 3 = no change, and 5 = a lot more; a = significant age difference (*p* < 0.05).

**Table 4:** Associations between parent demographics and parent-perceived changes in their child’s movement behaviours during the COVID-19 pandemic (May 2020; *N* = 151).

	Outdoor Physical Activity and Sport	Outdoor play	Walking, Wheeling, and Cycling	Indoor Physical Activity and Sport	Indoor Play	Household Chores	Television and Other Screens	Social Media	Non-Screen-Based Sedentary Activity	Sleep Quantity	Sleep Quality	Family-Based Physical Activity	Family-Based Sedentary Behaviour	Overall Healthy Movement Behaviours
**Parent Age**	−0.02	−0.03	−0.02	−0.10	−0.10	−0.04	0.13	0.15	−0.08	0.14	−0.03	0.06	0.10	−0.10
**Parent Gender ^1^**	0.16	0.21 **	0.08	−0.07	<0.01	0.10	−0.04	−0.05	−0.02	0.01	−0.19 *	0.11	0.05	−0.05
**Marital Status ^2^**	0.27 **	0.26 **	0.21 *	0.09	−0.06	0.19 *	−0.11	−0.11	−0.08	−0.03	0.09	0.26 **	−0.15	0.13
**Parent’s Education**	0.03	0.04	0.07	0.02	0.21 *	0.15	0.05	0.08	−0.01	0.01	0.07	0.08	0.05	0.07
**Parent Work Status ^3^**	−0.11	0.04	0.04	0.05	−0.08	0.12	−0.01	0.03	−0.04	0.09	0.13	0.03	0.02	−0.08
**Household Income**	0.12	0.20 *	0.14	−0.01	−0.04	0.18 *	0.04	−0.04	−0.13	0.06	0.13	0.18 *	−0.09	0.02
**Household (Dwelling) Type ^4^**	0.11	0.28 **	0.09	0.01	−0.12	0.10	< 0.01	−0.07	−0.16 *	−0.13	0.12	0.19 *	−0.08	0.04
**Family Distress**	−0.18 *	−0.16 *	−0.21 *	0.02	0.04	−0.10	0.10	−0.03	0.06	0.02	−0.14	−0.21 *	0.03	−0.01

^1^ 1 = man, 2 = woman; ^2^ 1 = single, 2 = co-habited; ^3^ 1 = unemployed, 2 = employed; ^4^ 1 = apartment, townhome, or semi-detached home; 2 = detached home; * *p* < 0.05, ** *p* < 0.01.

**Table 5:** Parent-reported changes in their ability to support their child’s healthy movement behaviours during the COVID-19 pandemic (May 2020; *N* = 151).

	Total	Children	Youth	Boys	Girls
	*N* = 151	*N* = 121	*N* = 30	*N* = 113	*N* = 38
**Changes in parent-perceived ability to encourage and provide support for their child’s healthy movement behaviours, *M* (SD) ^1^**					
Encouraged child to participate in PA	3.49 (1.13)	3.52 (1.12)	3.40 (1.22)	3.46 (1.14)	3.58 (1.13)
Played outside or did PA with child	3.35 (1.08)	3.37 (1.12)	3.27 (0.91)	3.31 (1.07)	3.47 (1.11)
Drove or provided transportation for child to do PA	1.68 (1.14)	1.64 (1.09)	1.87 (1.31)	1.74 (1.19)	1.51 (0.96)
Encouraged child to do chores	3.73 (0.82)	3.72 (0.85)	3.77 (0.73)	3.79 (0.76)	3.55 (0.98)
Encouraged child to stop sitting/watching screens	3.72 (1.12)	3.73 (1.14)	3.67 (1.03)	3.77 (1.08)	3.54 (1.24)
Encouraged child to sleep	3.43 (0.76)	3.44 (0.78)	3.40 (0.67)	3.43 (0.73)	3.42 (0.83)
**Changes in parent-perceived capability and opportunity to support their child’s healthy movement behaviours, *M* (SD)**					
Capable of supporting child’s physical activity	3.51 (1.03)	3.50 (1.00)	3.53 (1.14)	3.41 (1.04)	3.79 (0.93)
Opportunity to support child’s physical activity	3.54 (1.03)	3.50 (1.02)	3.70 (1.09)	3.42 (1.05)	3.89 (0.92)
Capable of restricting child’s screen time (e.g., screen breaks)	2.74 (1.31)	2.78 (1.33)	2.60 (1.22)	2.67 (1.29)	2.95 (1.35)
Opportunity to restrict child’s screen time (e.g., screen breaks)	2.64 (1.25)	2.64 (1.24)	2.62 (1.30)	2.61 (1.23)	2.71 (1.31)
Capable of supporting child’s sleep	3.90 (0.91)	3.90 (0.90)	3.90 (0.96)	3.95 (0.92)	3.76 (0.88)
Opportunity to support child’s sleep	3.89 (0.91)	3.89 (0.90)	3.90 (0.98)	3.95 (0.91)	3.74 (0.89)

^1^ Ranges from 1 to 5, where 1 = a lot less, 3 = no change, and 5 = a lot more.

**Table 6:** Associations between parental support and parent-perceived changes in their child’s movement behaviours during the COVID-19 pandemic (May 2020; *N* = 151).

	Outdoor PA and Sport	Outdoor Play	Walking, Wheeling, and Cycling	Indoor PA and Sport	Indoor Play	Household Chores	Television and Other Screens	Social Media	Non-screen-BasedSedentary Activity	Sleep Quantity	Sleep Quality	Family-Based PA	Family-BasedSedentary Behaviour	Overall HealthyMovement Behaviours
**Parents Encouragement and Support of Healthy Movement Behaviours**														
Encouraged PA ^1^	0.23 **	0.26 **	0.19 *	0.16 *	0.06	0.19 *	−0.07	−0.09	.05	0.09	0.06	0.26 **	0.17 *	0.17 *
Participated in PA	0.26 **	0.34 **	0.39 **	0.14	0.11	0.08	−0.03	−0.18*	−0.02	−0.01	0.04	0.41 **	0.21 *	0.06
Provided transport to PA	0.05	−0.06	−0.04	0.09	−0.21*	−0.25 **	−0.14	−0.02	−0.15	0.08	<0.01	−0.12	−0.11	0.11
Encouraged chores	0.04	0.07	0.02	−0.04	0.12	0.61 **	0.07	0.07	0.05	0.10	−0.07	0.02	0.17 *	−0.01
Encouraged reduced screens	−0.10	−0.13	0.04	−0.03	−0.13	0.10	0.19 *	0.06	0.05	0.20 *	0.13	−0.04	0.11	−0.08
Encouraged sleep	−0.04	< 0.01	−0.10	0.02	−0.04	<0.01	0.08	−0.12	−0.09	−0.03	−0.02	−0.07	<−0.01	−0.07
**Parent-Perceived Capability and Opportunity to Support Healthy Movement Behaviours**														
Capable of supporting PA	0.29 **	0.23 **	0.23 **	0.09	0.07	−0.04	−0.05	0.01	0.07	0.04	0.13	0.39 **	0.06	0.23 **
Opportunity to support PA	0.29 **	0.31 **	0.18 **	0.20*	0.16	0.01	−0.13	0.02	0.11	0.06	0.20*	0.35 **	0.08	0.37 **
Capable of restricting screens	0.42 **	0.33 **	0.28 **	0.13	0.17 *	0.17 *	−0.30 **	−0.20 *	0.08	−0.10	0.13	0.22 **	−0.05	0.20 *
Opportunity to restrict screens	0.35 **	0.29 **	0.22 **	0.02	0.10	0.11	−0.27 **	−0.07	0.08	−0.01	0.14	0.18 **	−0.02	0.21 *
Capable of supporting sleep	0.04	0.10	0.07	−0.18 *	0.05	0.19 *	−0.05	−0.07	−0.04	0.16	0.39 **	0.20 **	−0.04	0.12
Opportunity to support sleep	0.08	0.14	0.06	−0.16 *	0.10	0.21 *	−0.02	−0.10	0.02	0.19 *	0.41 **	0.21 **	−0.01	0.18 *
**Overall Parental Encouragement and Support and Parent-Perceived Capability and Opportunity to Support Healthy Movement Behaviours**														
Encouragement and support	0.14	0.15	0.17 *	0.12	−0.04	0.18 *	0.01	−0.08	−0.03	0.14	0.05	0.16	0.16 *	0.06
Capability and opportunity	0.38 **	0.35 **	0.26 **	0.04	0.16	0.16 *	−0.22 **	−0.11	0.07	0.06	0.31 **	0.37 **	0.01	0.31 **

^1^ = physical activity; * *p* < 0.05, ** *p* < 0.01.

## Data Availability

For more information regarding data availability, please email Sarah Moore, sarah.moore@dal.ca, and Kelly Arbour-Nicitopoulos, kelly.arbour@utoronto.ca.

## Appendix A

**Table A1:** Selected items from the parent-reported children and youth movement and play behaviours survey (adapted from Moore et al., 2020, and sent to participants from the National Physical Activity Measurement (NPAM) study to complete electronically using RedCap© in May 2020).

Survey Items	Response Options
**A.** **Screening Questions**	
Has anyone in your household been diagnosed with COVID-19?	Yes [thank you, terminate] No [continue]
Is your household under a self-isolation or quarantine order?	Yes [thank you, terminate] No [continue]
**B.** **Demographics (Parent)**	
Age	[age rollup]
Gender	[gender rollup]
Canada regions	[provinces, territories rollup]
Ethnicity	[ethnicity rollup]
Marital status	[marital status rollup]
Employment	[employment status rollup]
Education	[education rollup]
Household income	[household income rollup]
Household make-up (number of adults)	[number of adults rollup]
Household make-up (number of children)	[number of children rollup]
Type of residence	[type of residence rollup]
Residence postal code	Text field
**C.** **Demographics (Child)**	
Age	[age rollup]
Gender	[gender rollup]
Ethnicity	[ethnicity rollup]
Disability	[disability rollup]
**D.** **Child’s Current Movement Behaviours**	
In the last week, how many total hours and minutes per day did your child watch TV, use the computer, use social media, or play inactive video games?	Weekdays (per day) _______ Hours AND Minutes Don’t know Weekend (per day)_______ Hours AND Minutes Don’t know
In the last week, on how many days was your child physically active, so much that they were out of breath or sweating, for at least 60 min per day?	Drop down <<< 0–7 days >>>
In the last week, on how many days did your child engage in light physical activity for 2 or more hours per day?	Drop down <<<0–7 days>>>
In the last week, how many hours did your child usually spend sleeping in a 24 h period (including naps but excluding time spent resting)?	Dropdown <<<0–24 h>>>
**E.** **Change in Movement and Play Behaviours**	
Compared to before the COVID-19 outbreak and related restrictions, my child walks or cycles in the neighbourhood:	A lot less A little less About the same A little more A lot more
Compared to before the COVID-19 outbreak and related restrictions, my child is doing physical activities or sport outdoors:	A lot less A little less About the same A little more A lot more
Compared to before the COVID-19 outbreak and related restrictions, my child is doing physical activities or sport inside:	A lot less A little less About the same A little more A lot more
Compared to before the COVID-19 outbreak and related restrictions, my child is doing household chores (e.g., cleaning, yard work):	A lot less A little less About the same A little more A lot more
Compared to before the COVID-19 outbreak and related restrictions, my child plays outdoors:	A lot less A little less About the same A little more A lot more
Compared to before the COVID-19 outbreak and related restrictions, my child plays inside:	A lot less A little less About the same A little more A lot more
BEFORE the COVID-19 outbreak and related restrictions, my child’s primary playmate was:	Themselves Sibling(s) Parent Other Caregiver Friend(s) Other [specify]
Now, my child’s primary playmate is:	Themselves Sibling(s) Parent Other Caregiver Friend(s) Other [specify]
Compared to before the COVID-19 outbreak and related restrictions, my child watches TV, movies, uses the computer for leisure, or plays sedentary video games:	A lot less A little less About the same A little more A lot more
Compared to before the COVID-19 outbreak and related restrictions, my child uses social media:	A lot less A little less About the same A little more A lot more
Compared to before the COVID-19 outbreak and related restrictions, my child does other sedentary leisure activities not in front of screens (e.g., reading, puzzles, crafts, music, or art):	A lot less A little less About the same A little more A lot more
Compared to before the COVID-19 outbreak and related restrictions, my child sleeps:	A lot less A little less About the same A little more A lot more
Compared to before the COVID-19 outbreak and related restrictions, my child’s sleep quality is:	A lot worse A little worse About the same A little better A lot better
Compared to before the COVID-19 outbreak and related restrictions, our family time spent doing physical activity is:	A lot less A little less About the same A little more A lot more
Compared to before the COVID-19 outbreak and related restrictions, our family time spent doing sedentary behaviours (e.g., watching TV, playing board games, doing crafts) is:	A lot less A little less About the same A little more A lot more
Compared to before the COVID-19 outbreak and related restrictions, the balance of my child’s overall healthy movement behaviours (i.e., physical activity, sedentary behaviours, and sleep) is:	A lot worse A little worse About the same A little better A lot better
As a result of the COVID-19 outbreak and related restrictions, is there an inside leisure activity or hobby that your child is doing a lot more now?	Yes [specify] No
As a result of the COVID-19 outbreak and related restrictions, is there an outdoor leisure activity or hobby that your child is doing a lot more now?	Yes [specify] No
As a result of the COVID-19 outbreak and related restrictions, has your family begun any new or novel activities not previously practiced?	Yes [specify] No
As a result of the COVID-19 outbreak and related restrictions, has your family used any online resources or apps to support healthy movement behaviours?	Yes [specify] No
As a result of the COVID-19 outbreak and related restrictions, has there been a decrease in your child’s health (e.g., existing condition worsened or new condition developed)?	Yes [specify] No
Using the scale below, please select the number that best describes how much distress your family has been experiencing over the past week.	0 (No Distress) 1 2 3 4 5 6 7 8 9 10 (Extreme Distress)
**F.** **Encouraging Child Movement Behaviours**	
Compared to before the COVID-19 outbreak and related restrictions, I have encouraged my child to participate in physical activity or sport:	A lot less A little less About the same A little more A lot more
Compared to before the COVID-19 outbreak and related restrictions, I play outdoors with my child or do physical activity with my child:	A lot less A little less About the same A little more A lot more
Compared to before the COVID-19 outbreak and related restrictions, I drive or provide transportation for my child to do physical activity or sport:	A lot less A little less About the same A little more A lot more
Compared to before the COVID-19 outbreak and related restrictions, I have encouraged my child to do household chores (e.g., cleaning, yard work):	A lot less A little less About the same A little more A lot more
Compared to before the COVID-19 outbreak and related restrictions, I have encouraged my child to stop sitting and watching screens (e.g., screen breaks):	A lot less A little less About the same A little more A lot more
Compared to before the COVID-19 outbreak and related restrictions, I encouraged my child to sleep between 9 and 11 h per night:	A lot less A little less About the same A little more A lot more
Throughout the COVID-19 outbreak and related restrictions, and assuming I am fully motivated, I am capable of supporting my child’s physical activity over the next two weeks.	Strongly disagree Disagree Neutral Agree Strongly agree
Throughout the COVID-19 outbreak and related restrictions, and assuming I am fully motivated, I will have an opportunity to support my child’s physical activity over the next two weeks.	Strongly disagree Disagree Neutral Agree Strongly agree
Throughout the COVID-19 outbreak and related restrictions, and assuming I am fully motivated, I am capable of restricting my child’s screen time to no more than 2 h per day over the next two weeks.	Strongly disagree Disagree Neutral Agree Strongly agree
Throughout the COVID-19 outbreak and related restrictions, and assuming I am fully motivated, I will have an opportunity to restrict my child’s screen time to no more than 2 h per day over the next two weeks.	Strongly disagree Disagree Neutral Agree Strongly agree
Throughout the COVID-19 outbreak and related restrictions, and assuming I am fully motivated, I am capable of supporting my child’s sleep over the next two weeks.	Strongly disagree Disagree Neutral Agree Strongly agree
Throughout the COVID-19 outbreak and related restrictions, and assuming I am fully motivated, I will have an opportunity to support my child’s sleep over the next two weeks.	Strongly disagree Disagree Neutral Agree Strongly agree
